# Communicating cystic fibrosis newborn screening results to parents

**DOI:** 10.1007/s00431-020-03829-8

**Published:** 2020-10-17

**Authors:** L. Seddon, K. Dick, S. B. Carr, I. M. Balfour-Lynn

**Affiliations:** Department of Paediatric Respiratory Medicine, Royal Brompton Hospital, Sydney Street, London, SW3 6NP UK

**Keywords:** Cystic fibrosis, Newborn screening, Breaking bad news

## Abstract

The way results of cystic fibrosis (CF) newborn screening are communicated to parents is critical yet is done differently across the globe. We surveyed parents of 101 children in our tertiary London paediatric centre with a 48% response rate. Parental responses were as follows: 40/42 (95%) said the information could not have been given over the phone and 39/43 (91%) said they wanted both partners present; 27/42 (64%) said it was helpful having the health visitor also present; and 37/40 (92%) felt it was acceptable to wait until the next day for the sweat test. We have reduced the time from first contact to arriving in the home to 2–3 h.

*Conclusion*: We believe that this survey backs up our approach of a home visit by a CF nurse specialist with the family’s health visitor to break the news. This is challenging in the current COVID-19 pandemic.

**Table:** 

**What is Known:** *• Breaking bad news can have a lasting impact on parents when not done the right way.* *• Giving results of cystic fibrosis (*CF*) newborn screening is done differently within the UK and around the world.* **What is New:** *• Our parental survey revealed that the majority (92%) believed this should be done face to face and not over the telephone.* *• There was a mixed response to whether the parents should be told the genotype (assuming the CF centre knew), and thus the CF diagnosis before the confirmatory sweat test was carried out.*

**Electronic supplementary material:**

The online version of this article (10.1007/s00431-020-03829-8) contains supplementary material, which is available to authorized users.

## Introduction

The UK cystic fibrosis (CF) newborn screening protocol uses the day 5–8 Guthrie card dried blood spot to measure immunoreactive trypsinogen (IRT). If the IRT is elevated > 99.5th centile, DNA analysis for 4 CFTR (CF transmembrane conductance regulator) mutations is measured, and in certain circumstances, a 2nd IRT measurement is made on day 21 (this is the IRT-DNA-IRT protocol) [1]. If only 1 mutation is detected initially, a further 50 gene mutations are tested. The screening laboratories inform the relevant CF specialist centre of a screen-positive result, and the centre contacts the parents to arrange a sweat test to confirm the diagnosis.

This time is traumatic for parents and has long-lasting effects on how they handle their child’s CF care, which can be improved with good communication [2]. Parents do not forget what was said and how it was communicated, particularly in this initial phase [3]. Internationally, CF centres differ in their approach [4, 5], with CF centres in 10/16 European countries making the first contact with parents by telephone [4]. In the UK, centres also use varying approaches, for example, structured telephone calls, direct contact by CF nurse specialists, contact by screening nurse specialists, or screening link health visitors. The UK national guideline does not specify who should contact the parents to inform them that CF is suspected [1].

Full details of our screening and communication process are available in our CF guidelines (www.rbht.nhs.uk/childrenCF). The CF nurse specialist visits the home with the health visitor to break the news, having phoned that morning to check the family are available. The sweat test is carried out the next day in the hospital with results available within the hour, communicated by a consultant and nurse specialist. The following week education of the family takes place with the full multidisciplinary team over 2 days (parents go home overnight). As part of our quality improvement initiative, we wished to obtain parental views on the process to see if it could be improved.

## Methods

An online survey with 52 questions (see online supplement) was developed and tested on two sets of parents for language and to ensure it would not cause upset. It was managed anonymously by Healthcare Communications UK Ltd. (https://healthcare-communications.com/). The electronic link to the survey was emailed in April 2017, with a single reminder 1 month later. It went to all parents of children diagnosed with CF or designated CF screen-positive inconclusive diagnosis (CFSPID) following newborn screening, from July 2007 until the end of 2016, as long as the whole process was handled by our centre alone. Ethics permission was deemed unnecessary by our Research & Development Department as this was a service evaluation. Throughout this paper, we use the term parents, but this refers to parents and/or carers.

## Results

Surveys were sent to parents of 101 children, and replies were received from 48 (47.5% response rate), although 2 started but did not complete the survey, and some did not answer all the questions (hence numbers do not always add up to 48). Children were aged up to 9 years (mean 4.3 years); 45 had a CF diagnosis, and 3 had CFSPID designation. Two families already had a child with CF. Full results are available in the online supplement, including individual parent comments.

### Initial telephone contact to arrange visit

- 32/46 (70%) said the health visitor should make the first phone call, but the other 30% could not think of who would be better to do this. 31/41 (76%) had met the health visitor prior to the call.

### Initial home visit to break news

- 40/42 (95%) said the information could not have been given over the phone. Of the 2 who said it could be done by telephone, one was glad it was face to face.
- 39/43 (91%) said they wanted both partners present at home, although this was only possible in 40/44 (91%). 8/41 (20%) said they would have been comfortable giving the partner the information.
- Waiting time from call to visit was 2–3 h in 15/46 (33%), 3–5 h in 20/46 (43%), and over 6 h in 11/46 (24%). 24/45 (53%) felt the timing was right, 19/45 (42%) too long, and 2/45 (4%) too short.
- 27/42 (64%) said it was helpful having the health visitor present, but some made quite negative comments.
- 37/41 (90%) felt the amount of information about CF provided was about right, 3/41 (7%) too little, and 1/41 (2%) too much.
- If the CF team knew the genotype (i.e. indicating the child had CF) at the time of the home visit, 14/34 (41%) said they would not want to know, 9 (26%) said they would, and 11/34 (32%) did not know.

### Sweat test process and diagnosis

- 3/40 (8%) felt that it was too long to wait for the following morning for the sweat test, but the rest felt it was acceptable.
- 36/39 (92%) said enough information was provided by the consultant, 2/39 (5%) too much, and 1 (3%) not enough. 32/39 (82%) said they understood the information.

### Full education process (following week)

- 33/35 (94%) said the amount of detail was acceptable.
- 27/35 (77%) felt two consecutive days was acceptable, 7/35 (20%) too long, and 1/35 (3%) too short. 27/35 (77%) felt the same level of information could not be given in clinic or a 1-day visit.

## Discussion

Whilst, overall, satisfaction with the process was high, there were some issues that some parents commented on negatively which can be viewed on the online supplement. We have made changes where possible, some are outlined in the discussion below. We accept this is all that the parents knew, so they could not compare with other processes, apart from the hypothetical question about face to face vs telephone for breaking the news. Additionally, we have no information from parents of children who turned out not to have a CF diagnosis. We also accept that the responders (response rate 48%) may have been those with more positive memories of the communications.

Parents had a strong opinion in favour of face to face, and we feel equally strongly about this, although accept across Europe this is not a standard practice [4]. Their numerous reasons and comments why can be found in the Online Supplement. Although we are only telling them the screening test indicates a further test for CF is required, we find that they almost all assume that their child does have CF, even though 15% turn out not to have the condition [6]. In the current COVID-19 crisis, we are unable to go into the home but still conduct the subsequent education visit face to face. Many centres in Europe, the USA, and Australia tell parents that their child may have CF by telephone [4, 5, 7]. One US study found that more distress and uncertainty were caused by informing parents by telephone or leaving answerphone messages [8]. Another US study found that parents of babies with CF prefer face-to-face contact, whereas those with congenital hypothyroidism supported telephone contact, presumably reflecting the outcomes of these two conditions [9]. An Australian study also found that parents favoured face-to-face communication of screening results [7]. On the other hand, a survey in Switzerland [10] where all parents are first contacted by telephone found that 74% were satisfied with information given over the phone, and although 78% were troubled or anxious after the call, it is likely as many would have been after a home visit. Face to face allows partners to be present, non-verbal clues to be taken account of, and communication skills to be used fully.

The advantage of the health visitor also being there is only relevant if they are already known to the family. Health visitors in the UK are registered nurses or midwives who undertake further 1-year training in community public health nursing. The most important aspect though is to have someone who is an expert in the condition so they can answer the multitude of questions. In many units, especially in the USA, it is the primary care physician (general practitioner equivalent) who makes that call, and this can be problematic [5]. Since this questionnaire, we now provide a script for the health visitor to use when phoning to say they and the CF nurse are coming to the home. We still feel this initial call is better than just arriving at the door unannounced, especially as we want the partner to be there.

Timing is critical, and the wait for the nurse and health visitor to arrive causes much anxiety, so we have managed to cut this down to 2–3 h. Waiting for the sweat test is even worse. One US study found that most parents experienced high levels of emotional distress waiting for this, and surprisingly, the median wait was 7 days [8]. A survey in Germany found that 78% of parents found a wait of 3 days for the sweat test too long [11]. We always carry out the test the next morning and plan the home visit to ensure this is always possible (thus we would not visit the home on a Friday).

The issue of what to say when we already have a diagnostic genetic diagnosis is difficult, as in theory, the blood spot could be wrongly labelled, so a sweat test is still needed as per national policy [1]. If asked directly if the nurse knows if their child definitely has CF and even when we have 2 genetic variants identified, we say confirmation of a diagnosis is still needed with the next day’s sweat test. When asked whether we should tell them the genetic result in the home, parents were clearly divided, and a third did not have an opinion. We now tailor the discussion at the home visit to take into account when we have a definite genetic diagnosis and if we know it is likely to be a child with CFSPID.

In conclusion, parents backed up our belief that face-to-face communication by an expert nurse specialist was the right method of communication and that periods waiting for information and diagnosis should be kept as short as possible. This applies to any significant unwelcome news that paediatricians must tell parents.

## Electronic supplementary material

ESM 1 (PDF 536 kb)

## Abbreviations

CF: Cystic fibrosis
CFSPID: CF screen-positive inconclusive diagnosis
