# Supplementary material for: Communicating cystic fibrosis newborn screening results to parents
Source: Eur J Pediatr. 2020 Oct 17;180(4):1313–6. doi: 10.1007/s00431-020-03829-8 (PMC7567649; doi:10.1007/s00431-020-03829-8)
Supplement: Supplementary file 1 — (PDF 536 kb) [file 431_2020_3829_MOESM1_ESM.pdf]

Communicating cystic fibrosis newborn screening results to parents

Seddon L, Dick K, Carr SB, Balfour-Lynn IM.

ONLINE SUPPLEMENT

Methods: questionnaire

Survey on the New Born Screening Process

Dear Parent(s),

We are writing to you because you and your child have been involved in our Cystic Fibrosis Newborn Screening (NBS) process. The Royal Brompton Hospital (RBH) has been involved in this work since 2007. We are always keen to find ways to improve the service we provide to our patients and their parents. To this end, we invite you to participate in our survey, which focuses on how parents are informed about NBS results and their initial contact with the RBH Cystic Fibrosis Team.

Currently the process includes:

i. Health visitor phone call
ii. Home visit - CF clinical nurse specialist and (usually) Health Visitor
iii. Sweat test visit to confirm the CF diagnosis (and discuss treatment to start immediately e.g. Creon)
iv. Two-day education visit at RBH meeting the multi-disciplinary team
v. First outpatient clinic appointment at The Royal Brompton
vi. Shared care (if applicable)

Since you have first-hand experience of this process we would be grateful for your feedback. We would like to hear about your personal experience of the NBS process, as well as your suggestions about what the RBH team can do to better support parents at this difficult time. We would appreciate feedback from each parent (if appropriate).

You are welcome to complete the attached questionnaire anonymously or to add your name. If this survey brings back memories you find you want to talk further about, you are also welcome to give us a call to discuss this.
Please answer the questions below and feel free to include any further comments and suggestions that come to mind. Your participation is much appreciated.

Please circle where appropriate and leave comments. If you can not remember then please put can not remember:

Demographics/background information

1) Are you : mother/father/prefer not to say/other

2) Name of your child and age at present (optional)?

3) Child's gender: m/f

4) Your age: (please circle)
Mum <20 <20-25 <26-30 <31-35 <36-40 <40- 45 >45

Dad <20 <20-25 <26-30 <31-35 <36-40 <40-45 >45

Other/ prefer not to say

5) What borough or county do you live in? (optional)

6) What year was your child diagnosed?

7) Are you a parent of more than one child with CF? Y/N
If Y-were they both born since 2007?

8) How many heel-prick tests were done? 1/ 2 / can't remember?

9) Do we see your child in a usual CF Mon/Fri clinic or a Wednesday Professor Davies clinic?

10) Do you attend a shared care clinic? Y/N Please specify?

First phone call from Health Visitor

10) Had you met your health visitor before the initial phone call to arrange the home visit with the CF Nurse? Y/N

11) Did the health visitor advise you that your partner should be present for the visit from the nurse specialist? Y/N

12) If so was this possible? Y/N Was this what you both wanted? Y/N

13) How long was the gap between the phone call and the visit? (please circle)
2- 3 hrs 3- 5 hrs > 6 hrs

14) Do you think the gap between the call and the visit was (please circle)
Too long /too short / just right

15) Was the health visitor the right person to make the initial call? Y/N
(please comment)

16) Can you think of anything we could have done differently to improve this part of the process?

Home visit by CF nurse and Health Visitor

17) How many weeks old was your child when the home visit by the CF nurse and health visitor took place?

18) How long did the visit take?
19) Which family member / friend(s) were present at the initial home visit?

20) Did you think the recommendation to have your partner home was helpful? Y/N

21) Do you think you would have been comfortable giving your partner the information yourself? Y/N

22) If you partner was unable to be with you how did you feel passing the information to your partner?

23) On the day did you feel you understood the purpose of the home visit? Y/N

24) Was it helpful to have your health visitor there? Y/N

25) Did you feel the level and amount of information about CF provided at the home visit by the CF nurse was (please circle): Too much / too little / about right

26) Was there any information discussed at the home visit which, in your opinion, should not have been touched upon at that time?

27) Was there anything more you would have liked to have been discussed?

28) Do you think that the information given at the home visit could have been given over the phone? Y/N
Please explain your answer:

29) You should have received written information on the day, did you read any of the following and was it useful?

- CF Suspected leaflet Y/N Useful Y/N/no opinion
- CF nurse letter Y/N Useful Y/N/no opinion
- Sweat test leaflet Y/N Useful Y/N/no opinion
- Map of the hospital Y/N Useful Y/N/no opinion

30) Were you advised not to look online prior to the sweat test? Y/N

a)Did you look online? Y/N

b) was it helpful? Y/N

c)Did you use the CF trust website as directed by the CF nurse at the visit? Y/N/other

Comment

30) Did you feel the home visit helped prepare you for the planned sweat test
visit/result the following day? (please circle)
Completely / partially / Not at all

Comment……………………………………………………………………….

Visit to RBH for Sweat Test

31) Was the time waiting for the sweat test (please circle): too long / too short / acceptable?

32) Did you first meet the consultant (please circle): before/ during / after the sweat test?
Was this right for you Y/N
If No would you have preferred before/ during/ after?

33) Was enough information provided at this time by the consultant?
Too much / too little / about right

34) Did you understand the consultant's information about Cystic Fibrosis at the time?

35) Did your child start any treatment at the sweat test?
Creon Y/N
Antibiotics Y/N

36) If so did you feel you had enough training to feel comfortable in giving this treatment? Y/N

37) Can you think of any ways this part of the process could be improved?

Education visit at RBH the following week

38) Did you see the same consultant or nurse at the sweat test and at the education visit? Y/N

39) Was the level and pace of information provided acceptable? Y/N

40Was there anything you would have wanted discussed that was not included??

41) Was a two-day visit too long / too short / acceptable?

42) Could you have had the same level of information given at a clinic visit or a one-day visit?

43) Would you have preferred the two-day education visit to be spread over a week?
e.g. Tuesday one week and the following Monday the next Y/N

44) Are there any other improvements you think we could make to this part of the process?

First Clinic appointment

45) Did you see the same consultant, Dietician, Nurse or Physio at your first clinic appointment? Y/N

If No would this have been helpful Y/N

46) Was the first clinic appointment too soon/not soon enough/ acceptable?

47) Do you have any suggestions for improvements that could be made to this part of the process?

Genotype

45) Do you know your child's CF genotype?

46) Can you remember when you were first told?
sweat test/education visit/ first clinic appointment /later/ other/?

47) Given that the sweat test is still recommended to *confirm* the diagnosis of CF would you have liked a member of the CF team to have informed you about the genotype detected from the heel prick test if known before the sweat test?

48) Are there any other comments about the NBS service provided at the Royal Brompton Hospital that you would like to make?

Shared Care

49) If you do shared care when were you first seen at your shared care centre?

50) Was this at a shared care clinic with the Brompton CF Team or just the shared care team?

51) Was this too long/ too short/ acceptable

Finally, Lasting Impressions

52) What is your overall impression of your family's first contact with The Royal Brompton's CF Team?

Thank you again for participating in this survey. Your responses will help us to further our continuing aim to provide outstanding care for our patients and their families.

Results

Demographics

Are you: Mother/Father/Other

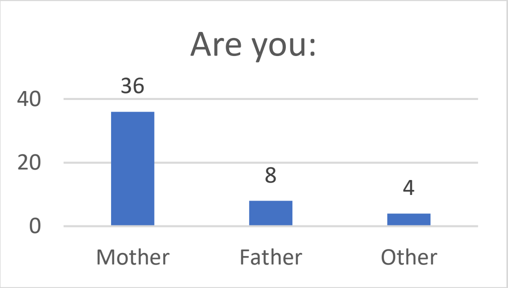

Respondee	Count
Mother	36
Father	8
Other	4

Current age of child:

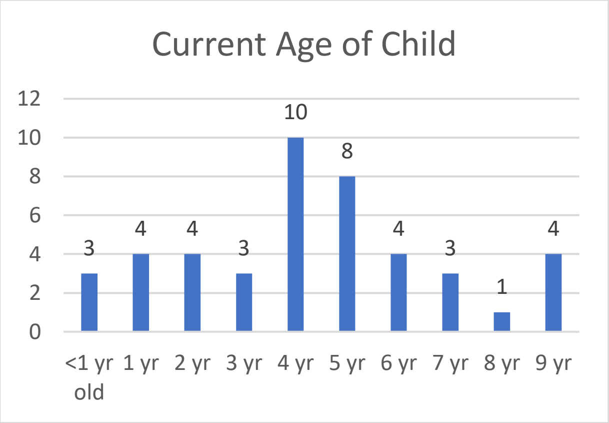

<1 yr old	3
1 yr	4
2 yr	4
3 yr	3
4 yr	10
5 yr	8
6 yr	4
7 yr	3
8 yr	1
9 yr	4

What year was your child diagnosed?

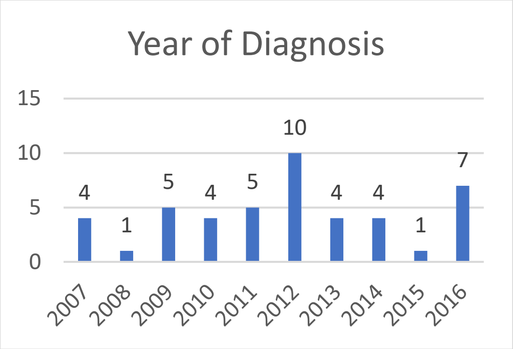

Year	Count
2007	4
2008	1
2009	5
2010	4
2011	5
2012	10
2013	4
2014	4
2015	1
2016	7

Are you a parent of more than one child with CF?

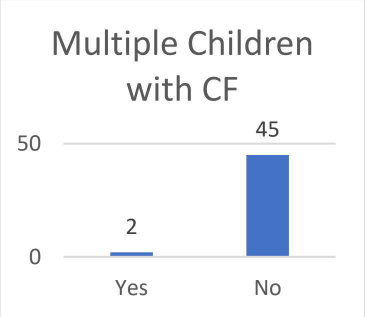

Yes	2
No	45

Had you met your health visitor before the initial phone call to arrange the home visit with the Nurse Specialist?

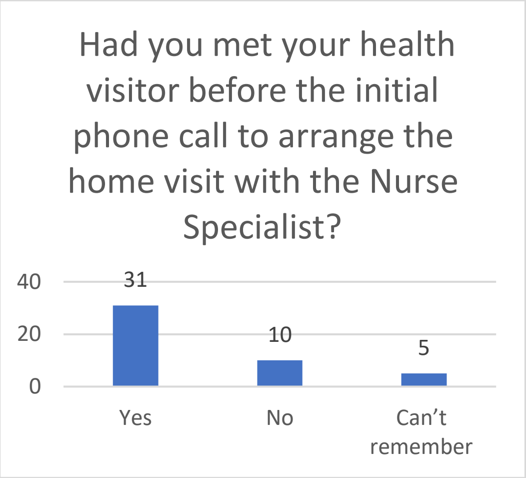

Yes	31
No	10
Can’t remember	5

Was it the same health visitor that came to deliver the screening result with the CF Nurse?

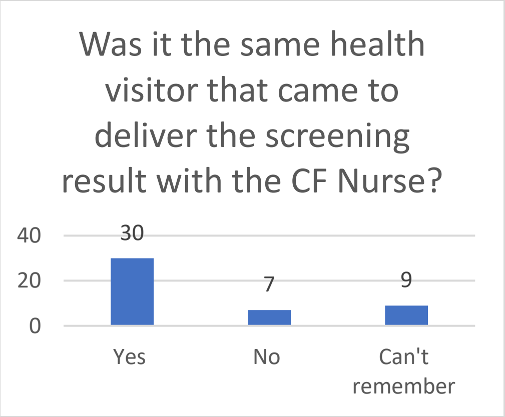

Yes	30
No	7
Can't remember	9

Did the health visitor advise you that your partner should be present for the visit from the nurse specialist during the telephone call?

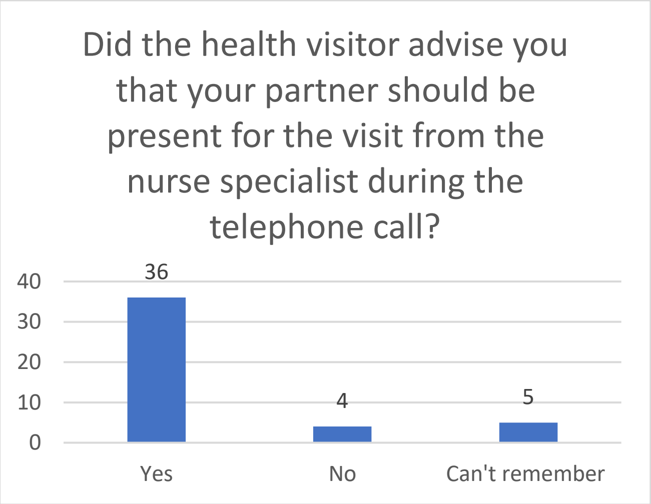

Yes	36
No	4
Can't remember	5

If so was this possible?

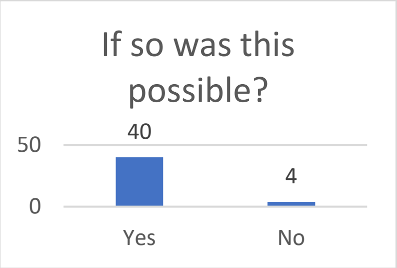

Yes	40
No	4

Was this what you both wanted?

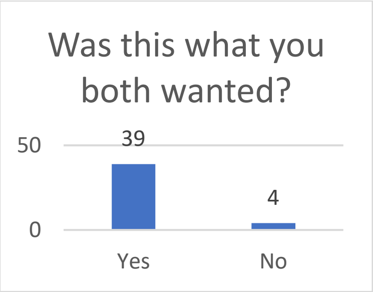

Yes	39
No	4

How long was the gap between the phone call and the visit?

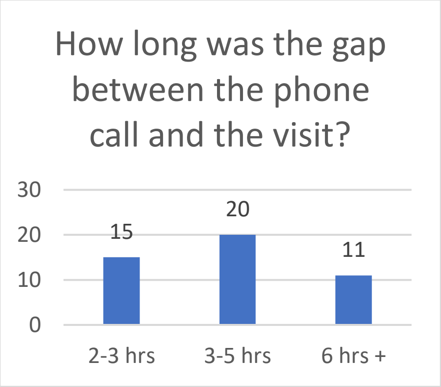

2-3 hrs	15
3-5 hrs	20
6 hrs +	11

Do you think the gap between the call and the visit was?

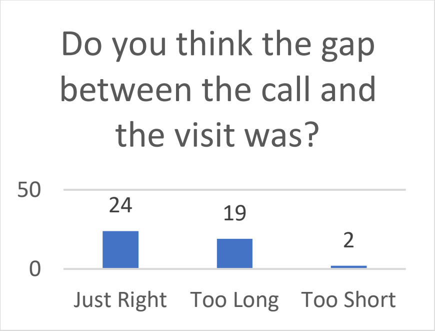

Just Right	24
Too Long	19
Too Short	2

Was the health visitor the right person to make the initial call?

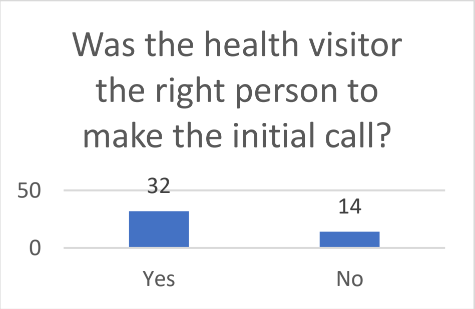

Yes	32
No	14

Comments received:

A call from the Health visitor alarmed me as it was made clear that something had come up on the heel prick test, however it would possibly have been more alarming to have come straight from someone from the Brompton.
Already felt comfortable with this health visitor so happy to have her present
As we had met the health visitor before it made the visit easier rather than a complete stranger calling to say they was coming around.
Comments: Age of mother/father completed above to reflect age at time of *** birth (rather than today) - question could be clearer It was possible for both *** mother and I to be home due to the quick turnaround of the heel prick test (ie since I was still on paternity leave) - this was helpful Thoughts about the call etc - I remember getting the call early in the day and the visit wasn't until quite late on; this period of uncertainty was very difficult. I don't know whether the HV was the right person to make the call or not but I do remember that the call was quite vague, which didn't help the uncertainty. (However, I appreciate that there's no really easy way to do this....)
Following the phone call you get a sense that there must be unhappy new to follow. I just froze in the time waiting for them to visit home and chose not to start internet searching about what the heel prick tests results could mean to us. I would have prefer the call just as they are about to visit with no thinking time in between. I understand this may not be possible as they have to plan their workload and day.
Getting a call from a stranger would have been even more daunting however waiting 4 hours knowing something is wrong but not knowing what is terrifying. I don't know what else could be done however as the specialist nurse had to get to us from London but it is a scary few hours when you don't know what's wrong with your new born a baby
I can't remember exact details. The process between the first and repeat blood test was not very good, and the information sporadic. I got a call to say the new born screening result came back inconclusive and needed repeating, a health care assistant who knew nothing about why she was re-taking the blood came to repeat. I asked her how long it would take and she said it should only take a few days.
I didn't actually have a phone call from my Health Visitor, I had to call them. This was actually the most upsetting part of the process as I received a text message from an unknown number saying 'please can you give me a call. HV' . It was just signed 'HV'. Now it seems obvious this stood for Health Visitor, but at the time, I left this txt for a few hours as I didn't think it was relevant to me and was possibly sent by mistake. I wish they hadn't abbreviated Health Visitor and had also more clearly outlined that it was in regards to my son, and was quite urgent.
I didn't know the HV very well - just recognised her face from the clinics I went to get *** weighed. She told me that she'd had a phone call about ***'s heel prick test and was waiting a call back and would let me know. It was the following day she turned up at home and told me that someone was coming to see me and that I should get my husband to come home.
I didn’t know why she was coming, didn’t expect to be anything serious. HV asked if my partner can be home and i said he can’t as he is at work. i was told she will be with another person, but she didn’t say it will be nurse specialist.
I had a call from the health visitor to say the first new born screening result as inadequate or inconclusive, therefore needed repeating. It was a health care assistant that came to do the test, I asked here a series of questions for which she did not know the answers to. I was advised it would take approx a week for the results. I did not hear back from the results after almost two weeks, I was advised the blood test went to ***, so I call the lab there to see if I could get the results. They advised they would only deal with the Health Visitor. I then called the Health Visitor the ask her to get in touch with them directly.

Within a few days the Health visitor called me to ask if she could visit with the nurse from the Royal Brompton.
I had met our health visitor on one occasion before the call. During that visit she had raised her eyebrows when I told her that Theodore wasn't gaining weight though he was feeding a lot. (I'm sure other CF parents have a similar experience.) She wasn't aggressive, but she did ask me to explain why he wasn't putting on weight, which made me feel a bit defensive. If I'd known why he wasn't putting on weight I would obviously done something about it. This meant I didn't particularly warm to her, and so the call coming from her was unfortunate in my case.
I had met the health visitor a couple of times, and can't think of anyone better who could of made the call.
I think someone from the RBH should of phoned and said your partner should be there when we come
It is a difficult call and I think it was better to hear from someone we had already met than a stranger.
It was all handled with such care. It was a complete shock to me and my husband. The Health Visitor called and within a couple of hours, the Health Visitor and *** (CF Nurse) came over and explained everything. They were both very caring and professional.
It was good to be called by someone I had previous contact with
It was good to get a phone call from someone you know
It was good to get the phone call from someone we knew
just about right drip feeding info at a very stressful time
Knowing the RBH nursing team as I do now, I feel it would have been more appropriate if they had made the initial call. Though I had met my health visitor before the call, it had been a brief visit and it's not as if we had a relationship. The health visitor was not at liberty to give any specifics during the initial call, which left room for a lot of fear and anxiety while we waited for the meeting.
My midwide came and told us she wanted to do another heel prick as there was something on the previous and she mentioned cystic fibrosis but told me this was really common and happened a lot so not to worry. However from the phone call from my health visitor to *** and *** arriving and the diagnosis the following day everything was dealt with perfectly and I wouldn't have liked it any other way.
Not in our case; we did not have a great relationship with our Health Visitor, but under the circumstances I cannot really recommend anyone else
The health visitor didn't know anything about CF so was really unhelpful. I am not sure who is the right person to make the call but I don't think a health visitor really needs to be at that initial visit unless they know more about CF. I found the follow up visits from the health visitor really unhelpful as her knowledge of CF was so poor and I found the visits a waste of time when I had so much else going on with a newly diagnosed baby.
The health visitor never made the call. It was made by the specialist nurse who came to visit us. The health visitor was definitely NOT the right person. She came to the appointment, but we had never met her before and we have never met her since or been contacted by her since. I found her presence at the time extremely voyeuristic as she merely sat and watched us go through an extremely painful and emotional torment and did not have any productive or positive role in that appointment or since.
The Heath visitor did not want to reveal the concern and did not know anything about CF.
To be honest it was all a shock and then was all a blur but they were very nice and made sure we understood what was being said

Was the worst phone call of my life.. makes me feel sick to my stomach when I think back to it and that's why I very rarely speak about it. She put the fear of death into me.. told me there is something seriously wrong with *** and that I needed to get my husband and family to come home immediately for her to come and see us with the hospital but she could not tell me over the phone what was wrong... would never want anyone to have to go through that.
We were understandably confused and anxious about why the health visitor was being so vague on the phone, considering the fact that it was obviously an important matter. However, we completely understand why you wouldn't wish to provide us with more information until the CF nurse was able to meet us in person.
When the Health Visitor called initially, she forgot to tell me that my partner needed to be present at the meeting - she called me back a short time after to tell me that "it would be a good idea to have someone to support you at the meeting" (these words have stuck in my memory as they made me worry frantically). The Health Visitor also wasn't confident at all in the situation - I understand my daughter was the first non-routine baby she had to deal with. Thankfully the CF nurse (***) who attended the meeting was excellent, and compensated for the Health Visitor's lack of experience and confidence.
When you receive a call saying they want to visit you immediately know something is wrong. The gap was too long leaving me knowing something wasn't right but not what it was - I got myself worked up into a state of panic in the time between the call & the visit
Would prefer my GP. Health visitor wasn't very helpful.
Yes although the gap was too short and hence extremely alarming. We had to rush home from hospital for it after it was moved back 24 hours.

Can you think of anything we could have done differently to improve this part of the process?

As from my comments above - a clearer message or a voice mail would have been much more appreciated. It seems such a small thing, but out of the whole day this is what sticks in my mind, as I was on the verge of deleting that text.
Better educate the midwives who are sent to re do the test and explain the importance of not brushing it off.. however I am fully aware that this was nothing to do with the Royal Brompton the cf team! Who are incredible every time !
Educate the health visitor on how to handle the initial first contact
For me it was the initial call stating they were even coming to talk about blood results and I spent the couple of hrs just thinking what it could be and got really upset and worried
I don't think people should be informed over the phone as it's a lot to take in and I understand the health visitor has to make sure you are available for a visit before the nurse travels from London so I'm not sure how the length of time between being told there's a problem and finding out what it is can be reduced but that is a really scary few hours
I think it might have helped if they said they suspected CF, I was just told a specialist nurse was coming
I think my was dealt with very well
I think that the phone call should be made and the parents told straight away what the concern is.

I think the process could have been faster, and more coordination between teams. Having a blood test repeated with vague reasons as to why it was being repeated was not very good. It increased the anxiety without proper information forthcoming. I think it took about 4 weeks in all to get the diagnosis which is inefficient in my eyes. I had to call *** hospital to chase the 2nd result as it was taking so long. I was told they would not speak to me but a health visitor. I think I may have called the health visitor the advise of this. I think the sample was there but had not been processed, I never got a full explanation at to why it took so long with the result.
In my experience the RBH nursing team manage to at once be sensitive and straightforward. I feel that if my first contact had been with them I would have felt less at sea in the horrible few hours between that first call and our meeting.
It was very difficult as the call came at 9am and I spent till 5pm worried sick.
It would have been good to know more during the phone call. The wait was too long and we wondered what was wrong with our child. Considering the phone call and the request for my husband to be at home for the home visit we knew something was wrong but didn't know what was wrong
More information regarding the home visit and shorter gap between phone call and home visit
No not really.. I have sat & thought long and hard but can't think of a better way of delivering this news to families.
No suggestions, other than possibly excluding the Health Visitor, as she didn't add any value to the process.
No, I think this was done correctly.
No, I think this was the best way, we didn't have to wait too long and it was good that our health visitor who we already knew was present.
No, it was all handled very well.
No. It was horrible.
No. The hospital was great. The health visitor less so.
Not have the health visitor present. Come round much sooner as by being told that someone is coming to visit you to discuss your baby's health highlights that there is something extremely wrong. Being made to wait to find out what it is was torment. I would much prefer to be given the exact information to process on my own then to be left waiting in limbo.
Not really - it was very difficult for the people delivering the news also & the gap between the call & the visit was necessary to get ***'s dad home in time. Although I was left feeling very uneasy & worried about what they were coming to talk through with me, I can’t think of a way that could of avoided this.
not really for me i like to have the info to hand so i can get on dealing with the issues, but not everyone is as pragmatic
RBH was really good
Shorten the time between call and visit. Maybe explain that the visit is to discuss further tests that are needed following the heel prick test.
Shorter gap between call and visit. 6 hours is too long
The Brompton's specialist CF nurses are incredibly sensitive, honest and kind. I would have preferred my initial contact to have been with one of the team.
The gap between the call and the visit should be longer, maybe even a day or few days so a partner can make work arrangements in advance. I also didn’t know the purpose of the visit, if i would have known, he would definitely took time off work.

The gap between the initial phone call and nurse specialist visit should be as short as possible. Not before a weekend.
The rest of the process was all fine.
Yes absolutely, the Health visitor could have made a note in her diary to chase up the results, I could have been kept more up to date and it would have been more appropriate that the person re-taking the blood knew the answers to my questions.

How many weeks old was your child when the home visit by the CF nurse and health visitor took place?

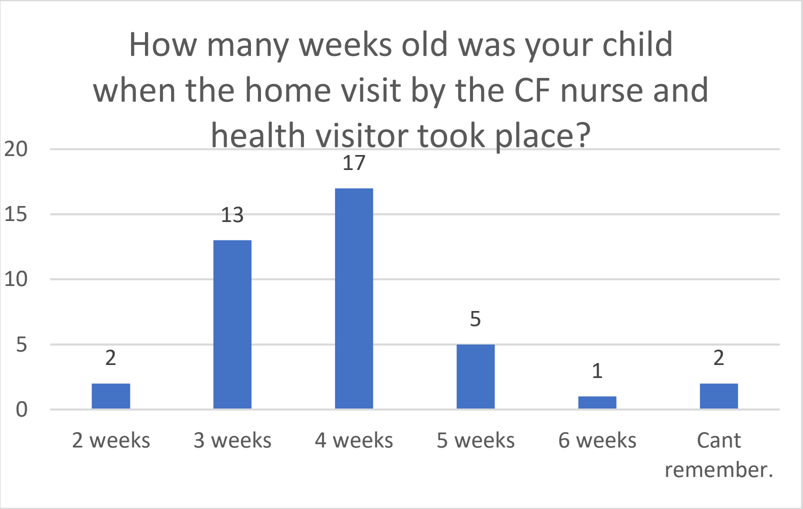

2 weeks	2
3 weeks	13
4 weeks	17
5 weeks	5
6 weeks	1
Can't remember	2

How long approximately was the home visit (in minutes)?

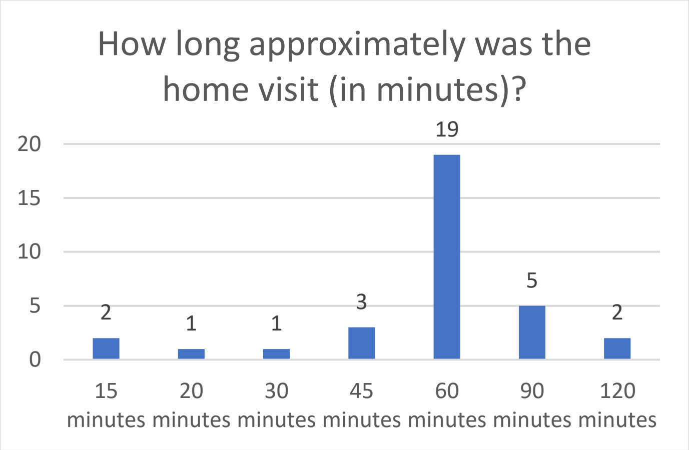

15 minutes	2
20 minutes	1
30 minutes	1
45 minutes	3
60 minutes	19
90 minutes	5
120 minutes	2

Which family member / friend(s) were present at the initial home visit?

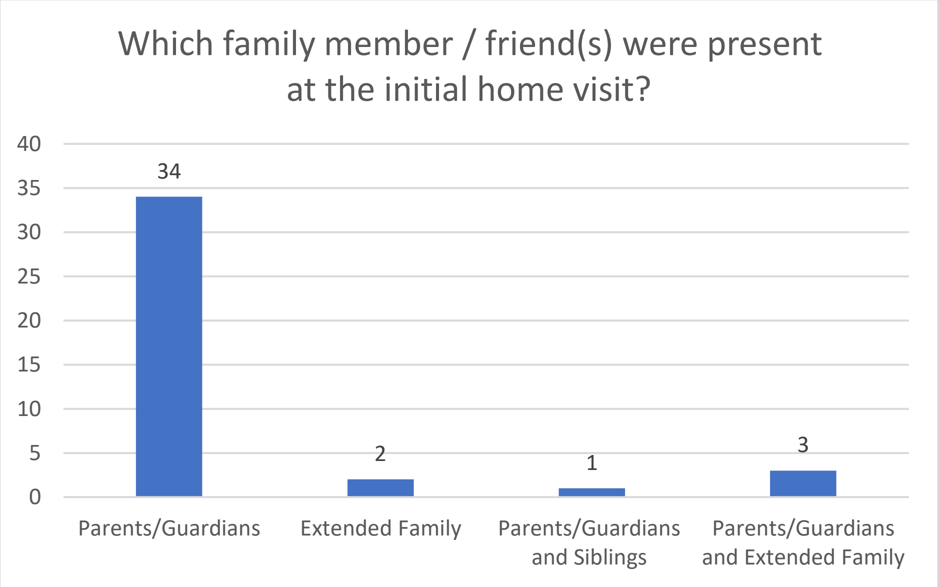

Parents/Guardians	34
Extended Family	2
Parents/Guardians and Siblings	1
Parents/Guardians and Extended Family	3

Did you think the recommendation to have your partner home was helpful?

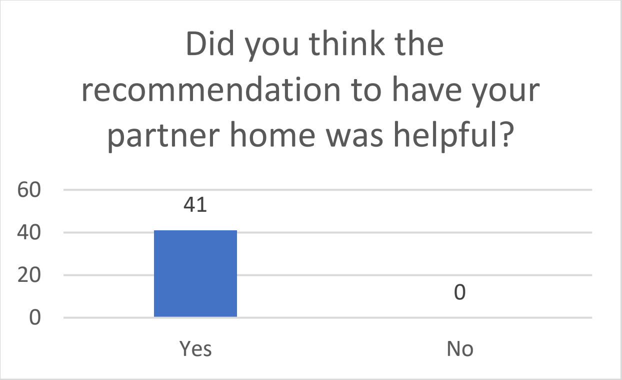

Yes	41
No	0

Do you think you would have been comfortable giving your partner the information yourself?

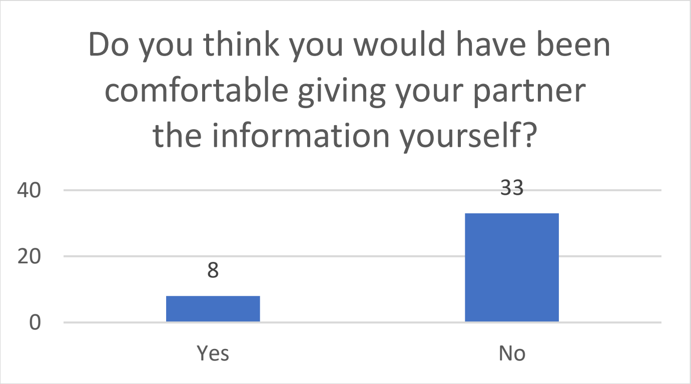

Yes	8
No	33

If you partner was unable to be with you how did you feel passing the information to your partner?

I feel it is such an emotive topic that I couldn't of explained it in a clear or calm enough manner. I also didn't know enough about CF to explain the facts to him as well as the CF nurse could
I think that would have been difficult.
I was referred to the CF website which had a lot of the information on. Otherwise I would have found it difficult to remember a lot of the info under the circumstances.
I was still shocked at the news, and just wanted him to be with me.
I would have been able to pass on some of the information to my husband, but he would have had a lot of questions and would have felt much more anxious had he not been there.
I would of hated it being told together is much better as we get all the information together instead of separately.
I would of hated it. I think 100% both parents should be there

It would have been extremely distressing. I don't think I could have delivered this. I know that immediately he would have had questions which either I would have not asked, and wouldn't have been able to answer. It was essential that he was there, it was just very lucky that my husband works from home.
It would have been fine but you both deserve to be told together
n/a
No comment
Wouldn't have had a choice but I don't know how well I would've been able to communicate the information as I'm not sure I took much information onboard after the initial shock

Was it helpful to have your health visitor there?

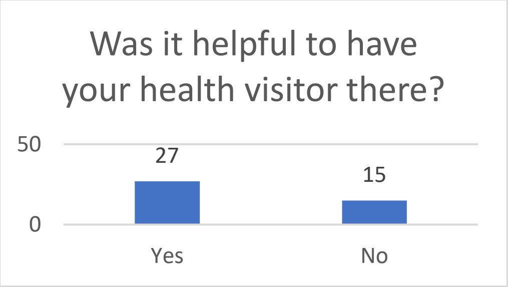

Yes	27
No	15

Comments received:

as mentioned before, the health visitor should NOT have been present at all. We had no relationship with her and she had no role or purpose then or at any time after.
because I had previously met her I felt slightly more comfortable with her there. she held my hand through what was a devastating time
Being honest I can't remember whether there was a recommendation to have both mother and father there or not - what I will say though is that it was definitely something that we would have wanted to go through together
Can't remember exactly, but hv didn't say much.
From what I remember, the health visitor was helpful as she was listening to all the information we were given on the day and then she visited us for baby checks at home over then next year. This may not have happened if she wasn't involved right from the start.
Health visitor seemed to know nothing and was of no real use at the visit
I already knew the Health Visitor, *** so it was nice to have her there.
It was good to have the support of someone we had met before
It was helpful to have the health visitor there because she knew us a little bit and it didn't feel like strangers coming to talk to us.
It was nice to have the support of someone we had met before
Looking back everything was done in the best possible way, however at the time it was all a blur.
needs a professional approach

Nice to have another person that was local that we could call upon if necessary but not knowing her that well meant that wasn't really the case. I recall her telephoning me a couple of times after the home visit to see how I was.
Our health visitor was nice, but I don't feel that she brought anything to the table.
The health visitor did very little, but it did seem helpful to have a visit from two staff.
the nurse was very very good and answered direct questions appropriately
Whilst the Heath visitor didn't add anything to the meeting seeing a familiar face was useful and they made sure I know I had their support at any time if I needed it
With the health visitor there it was a familiar face rather than *** who at the time was a complete stranger.
Yes it was helpful having the Health visitor there.

Did you feel the level and amount of information about CF provided at the home visit by the CF nurse was?

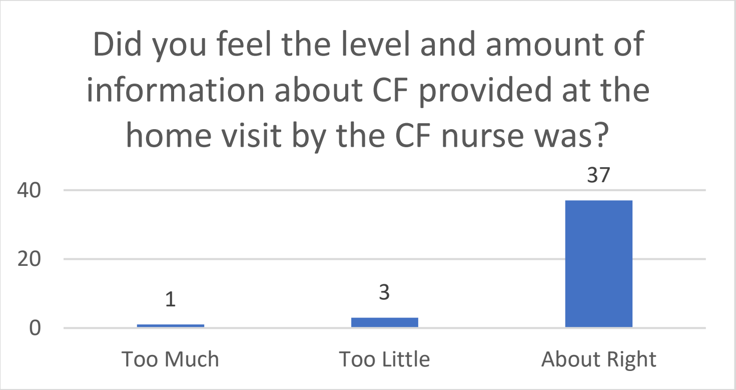

Too Much	1
Too Little	3
About Right	37

Was there any information discussed at the home visit which, in your opinion, should not have been touched upon at that time?

......all a bit of a blur I think i was way too upset to take any of it on board at the time
No
cannot remember (it's a blur as I was feeling devastated)
I can't remember what you told us at the home visit, other than our son has CF.
I think the only word that confused me at the time was 'suspected'. I felt that it possibly gave a little false hope that it could come back negative, even though the genetic strain had been found.
I think the right amount of information and advice was given. The nurse was very understanding but able to answer all my husband's matter-of-fact direct questions. She was superb.
I think what was difficult as they were not certain until the sweat test was done.
If I'm honest I don't remember much about the information given to me at the home visit.
It is hard to remember exactly but I don't think so.
Life expectancy was way too much info to take in at the time

No - we asked some hard questions (as in sensitive, not difficult) which were handled well Our philosophy is to be as transparent as possible (with ***, each other and the health workers) so no information is off limits for us really
No.
No. I think it's important you have all the facts straight away so you know exactly what you are dealing with
No. It was the right amount of information.
None
Not at all
not really
Not really just the initial life expectancy of cf patients
Nothing that sticks in my memory. I don't remember too much about the content of the meeting, other than that *** was excellent in delivering the news - direct and confident, yet very compassionate.

Was there anything you feel we should have discussed at the home visit that we didn't?

Bit more info about cf
cannot remember (it's a blur as I was feeling devastated)
Cant remember.
I cannot remember..
I don't honestly know.
I would have liked more information about testing for my daughter. Maybe even the sweat test to have been carried out there and then.
No
no - but you don't know at that stage what are the questions to ask
no but at that stage you have no idea what questions to ask
No from what I remember
No, it was just right.
No.
None
Not from memory

Do you think that the information given at the home visit could have been given over the phone?

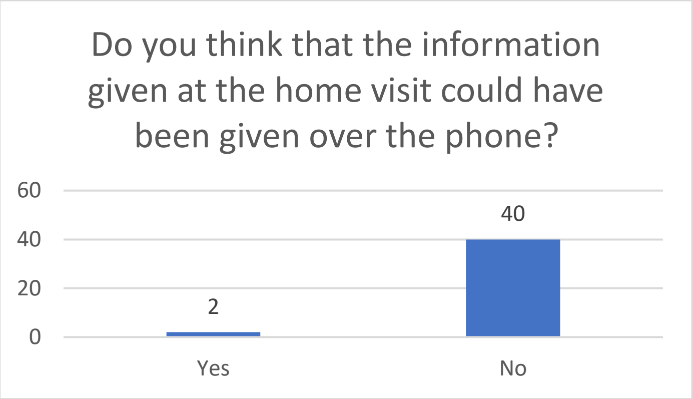

Yes	2
No	40

Please explain your answer:

Being told your child has CF is huge & I think the home visit & being told in person is the best way, face to face means conversation flows better & gives the opportunity to get the information you need
Content-wise, of course it could, but it definitely helped to have an expert in the room. Apart from anything else I'm not sure how a phone call would be structured - we spent a lot of time collecting our thoughts which is easier done in a face-to-face environment than on the phone
Devastating news like this needs to be given in person. *** gave us time to take it all in, she offered to leave the room, she allowed us time to think about any questions.
For me it was massively reassuring to have our specialist nurse there. The whole situation seemed so unreal - we thought our little boy was absolutely fine one minute and suddenly someone was telling us that he had a serious illness. If our nurse hadn't been there I'm not sure I'd actually have taken any of it in.
For something life changing like Cystic Fibrosis it needs to be done face to face.
I prefer face to face meetings, especially when it is such a information.
I would of been horrified if I was told over the phone
I would personally have preferred to have had the diagnosis on the phone then to be left waiting for ages to find out what the problem was. By the time the nurse and health visitor finally turned up I was an anxious mess and not able to process any of the information I was being given.
If I got that information by phone I would not know what to do as I would of been in shock and would not of taken it in having *** the cf nurse and the health visitor there helped make it more real and I absorbed it more
It could have been given over the phone but I'm glad it was done face to face.
It is poor clinical practice to deliver bad news over the phone. You would miss visual cues from parents.
it is something that needs to be discussed face to face, news like that cannot be given over the phone, it's extremely delicate

It is too big a diagnosis and shock for parents of a newborn to hear over the phone. It was invaluable to have a decent amount of time with a specialist if any less or over the phone I think we would have resorted to google.
It is very difficult news for a parent to hear. Personally, I broke down in tears, and I would not have stayed on the phone for long enough to have gained received all of the information that the CF team probably wanted me to hear. For me, it was good that I was given a few minutes to absorb and react to the news - these sorts of silences aren't very easy on the phone (the call would have probably been rushed as everyone would have felt even more uncomfortable!)
It needs to be in person as its a huge and devastating moment to know your child has a chronic illness and a limited life ahead, and not only this but you have been looking after a newborn who has trouble feeding and its exhausting, you need to talk to someone face to face.
It was most certainly better delivered in person, receiving that message over the phone would have meant no one would have been with me & only I would have been told.
It was such a shock to learn that your newborn has a life-limiting condition - I think this can only be given in person by medical staff who are knowledgeable.
It was very important having a face to face conversation with my husband and our health visitor present
It's too huge to tell someone over the phone. There are too many questions that come out during the face to face discussion. A phone call is too impersonal for this type of news
It's very traumatic. Definitely a discussion in person was essential. Much more personal
My husband and I both experienced some amount of shock, so I think it's essential to be given this information in person.
needs face to face interaction some parents might need a lot more support
No, if the information was given out on the phone I would have been left with a million unanswered questions and I would have been given the information that I would then have to give to ***'s dad myself and got confused or upset.
not personal enough and some persons might take the news in a different way and need physical support on finding out about CF
On receiving the information I was very shocked and tearful. I would not have been able to speak clearly on the phone. Also in person they were able to give me a moment to gather my feelings and then carry on explaining . This would not have been as easy if not face to face.
Perfectly dealt with
The diagnosis was such a shock, having someone answering questions in person is vital. This is a life changing diagnosis, it needs to be given in person. We are not talking about telling a parent that their child has chicken pox
The support of the CF specialist nurses present in our house was invaluable. If discussed over the phone then it would have been much harder.
Things like this are much better face to face to give parents time to come to terms with the news.
This diagnosis is a life changing diagnosis so face to face visit is vital Parents need the reassurance and support
This is definitely information that needs to be delivered face to face. We had no suspicions that anything was wrong with our son (he hadn't lost any weight, and was growing normally), so this was a real shock to us. Our CF Nurse gave us time to cry and was very reassuring and in between telling us information - over the phone this would have felt extremely awkward.

This is very sensitive information and to relay it over the phone would be insensitive to the seriousness of the situation.
You're basically explaining that your newborn child will almost certainly die young from a horrible disease. Not great news to get over the phone.

Comments received about literature given:

Again, it was helpful to have something tangible.
All the leaflets were useful. You go through stages of sadness and then practicalities of how to actually get to the hospital and which area and you need all the leaflets to read as and when you feel like it.
Apologies - this might not be the right place to write this - but the lack of car parking at the RB was very distressing. it just added extra pressure that I felt was necessary. The car park operator was rude, and not at all supportive - and I have to enter the hospital on my own without the support of my husband while he had to try and find a place to park. It was probably one of the most distressing times attending an appointment to determine the health of my baby - and something as little and silly as car parking (just for that one day) really exacerbated the situation.
As the diagnosis was a shock, being able to read more after the visit is important
Cf suspected was too scary.
From what I can remember the information was fine.
I cannot remember much about actual leaflets been given, possibly information about the sweat test was given on the day... I don't remember any information been given before.
I can't remember having written information.
I remember the information was useful & I referred to it for weeks after & shared with my family to read. However I can’t remember what the information was exactly.
It took a few hours for us to look at everything.
It was OK at the time but was still in shock
Most useful piece was the nurse's letter and the contact details of both *** and ***. Perhaps the only comment I'd make is that my expectations of the sweat test could possibly have been managed better. Having read the CF suspected leaflet, I was clinging on to the word "suspected" and left feeling hopeful that the sweat test would actually come back with a negative result (i.e. a totally different conclusion). But on arrival at hospital, *** made it clear to me (in an effective way) that the heal prick test was highly unlikely to be wrong and that the sweat test was more of a formality. Perhaps I chose not to hear this at the initial home visit and I was clinging on to hope. But that made the beginning of the educational visit quite upsetting for me.
No written materials received
The diagnosis was a shock and we were given a lot of information. We didn't remember everything that was discussed so it is good to be given some written information to go through later when the shock has passed
The home visit and the sweat test were the day after each other so not much time to read and absorb any information really.

Could they have been improved?

Can't remember

Coming into hospital for the first time was overwhelming. We didn't know where to go for food or drink, we felt like we couldn't leave the bedside, we didn't find out about the parents’ facilities on the 6th floor for about a year and that was only after another parent told us. We didn't know where the local shops were or places we could eat. We asked about this at the time but nothing happened. I remember one nurse making a joke about CF (she said "Ahhh, is your baby crying because he has CF"). Well, I think it was supposed to be a joke. I can't imagine what else it would have been. It was incredibly upsetting and hurtful. The nurse still works on Rose Ward and I see her every time we come in for an admission.
Don't remember
I think they need to just give basic information, I wouldn't have taken much info in at that point anyway
I though the leaflets were fine.
No
No - they were very good
No I think it covered everything we needed to know
No information was clear
no they were good.
No.
Nurse was brilliant and very clear. Helpful in arranging parking as I couldn't walk at this stage as a result of pregnancy complications.
Possibly yes.
See above..... the word "suspected" in the "CF Suspected Leaflet" misled me and gave me false hope. Admittedly I haven't re-read the leaflet since, so this confusion may well simply have been a result of me being an exhausted, first time mum!
To be honest, I don't think we actually read them at the time as we were shell shocked. They were helpful to have though and I carried them with me to the first appointments at the hospital.

Were you advised not to look online prior to the sweat test?

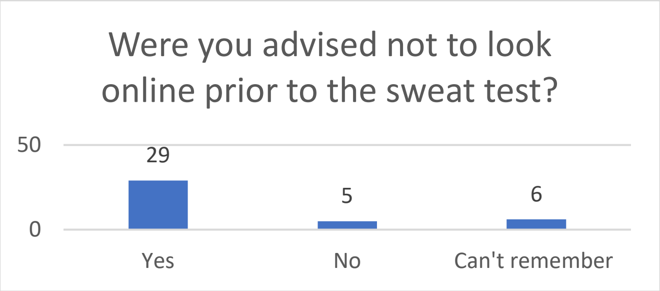

Yes	29
No	5
Can't remember	6

Did you look online?

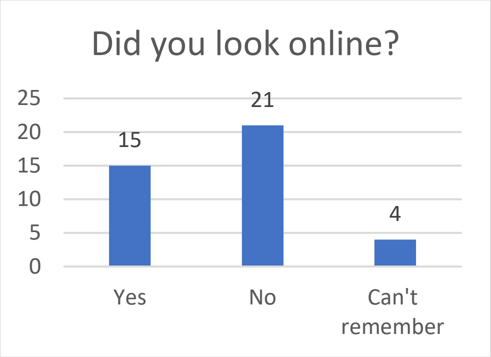

Yes	15
No	21
Can't remember	4

Was it helpful?

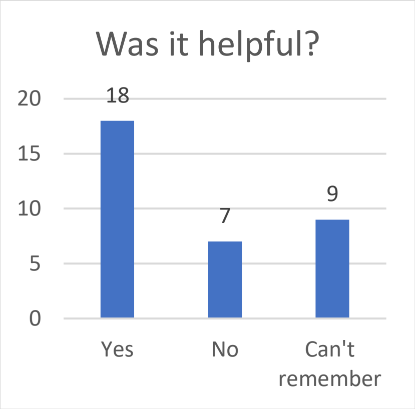

Yes	18
No	7
Can't remember	9

Did you use the CF trust website as directed by the CF nurse at the visit?

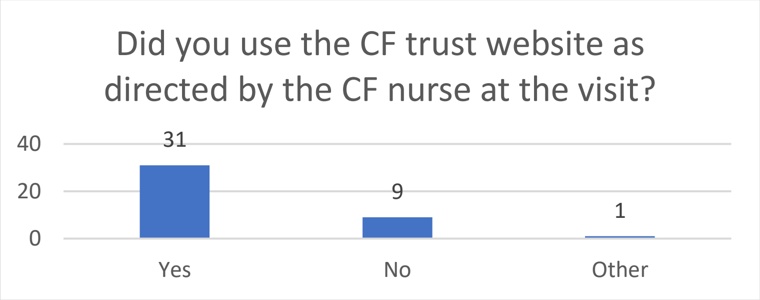

Yes	31
No	9
Other	1

Comments Received:

As soon as I got the call to say the health visitor & one other were coming to discuss the sweat test I started to google what the sweat test was for to see the possible reasons.
But at that time information was very out of date e.g. Life expectancy and they did not have the new parent pack.
didn't look on line and it was some considerable time before looking at CF trust site
didn't look on line and waited a good year before looking at CF trust site
Didn't look online. Still don't really!
I decided to use the time I would have been googling to write a list of questions for the education days. Family members have since told me they did go & look online & it wasn't helpful & just upset them
I didn't believe my baby had anything wrong with him so didn't feel the need to look at anything (was totally in denial)
I didn't look myself but my husband did and read a few bits out to me.
I felt like we were being given a kind approach to the illness and i didn't want to turn up to the hospital to find out that CF was much worse than i thought. I felt like i wanted to be fully and emotionally prepared to listen to what the doctors were going to tell us and not just be left reeling from shock.
I remember being advised not to do internet searches, and instead to look only at the CF Trust (I can't remember at which meeting we were directed to the CF Trust website). Likewise, I was advised to ask my family and friends not to do lots of internet searches and instead to look only at the CF Trust website - I think this is excellent advice, and this is something that I still stick to.
I think not looking online is good advice as it can be very upsetting
I think that we were advised to only look at the CF trust website and nowhere else - this is sound advice
I took the advice to not look online very seriously. I still don't look five years later. The only time I did look was after the initial call from the health visitor and before the home appointment with our specialist nurse and the health visitor. It was the worst thing I could have done as it made me extremely anxious.
I will not look on line don't want to know the horror stories would rather as the professionals
It was a very difficult time but I feel like it was handled in the best possible way with regards to the visit, documents etc.
Just to clarify- we were advised to look online BUT only at CF Trust website and not to look at other sites online so we followed this advice.
Of course we Googled CF the minute they left!
The CF website in terms of info is fine, but the forum was fear inducing as people access it in times of distress and I found this extremely upsetting
There are some really promising stories on there but I did not look in any detail as don't want to see bad stores either
We contacted the cf trust for a newly diagnosed pack after we had taken our son for the sweat test. I am thankful for the advice to only visit the cf trust site. It is without doubt the most informative, supportive and up to date.
We wanted to speak to the consultant first and have the confirmation that our child had CF first
We wanted to wait for the visit at hospital before looking at more information regarding cf
Yes just looked at the CF trust website

Did you feel the home visit helped prepare you for the planned sweat test visit/result the following day?

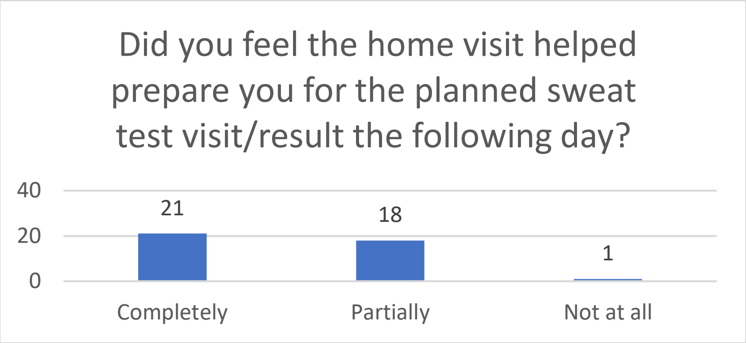

Completely	21
Partially	18
Not at all	1

Comments received:

Everything was happening so quickly, that didn't have much time to research online. But i don't think i could prepare for the sweat test any better. You would just never be ready, just have to do it.
for me it was what i was and i needed to get on with it to ensure *** had the best treatment
I don't think anything can fully prepare you for the enormity of what it means or what you need to learn very quickly
I don't think much can prepare you, especially as we had no idea.
I don't think the news that your child has a life limiting condition can ever be received in anything but a negative light. I am not sure there is a 'right' way that will ever tick all the boxes. I do think the specialist nurse was as sensitive as possible and gave as much info as possible.
I knew what was going to happen when I got there and it helped us
I learned a lot more on the sweat test day but that is because we had time to digest the news and think of further questions we wanted to ask
It was a lot to take in
It was good to have being information before the visit at hospital Going to hospital is always daunting
It was good to meet a member of staff from the Brompton in our home setting and we felt well prepared for the visit the next day.
It would not have been possible for us to feel "prepared" but it did help.
Nothing could have prepared us.
Our CF nurse, ***, was exceptionally kind, understanding and reassuring. I can't imagine anyone doing a better job.
Our nurse talked us through the process, so we were fully prepared for the visit.
See above - I was still clinging on to hope that the sweat test was the "real" test, and that there was a chance that it could come back negative. However, this may be due to my selective hearing and me being in denial, as opposed to lack of information being communicated to me - I can't really remember.
Was still a lot to take in
We were in shock and didn't realise what the sweat test/result visit would be

Yes (just a bit of a hard time processing all the info so I don't think I ever would have felt fully prepared, that's all)
Yes, we knew what was going to happen, where to go and what to expect, it was explained to us very well.

Was the time waiting to come to the Brompton hospital for the sweat test?

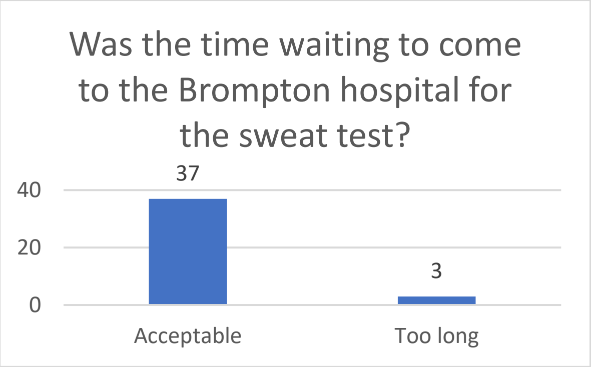

Acceptable	37
Too long	3

Was enough information provided at this time by the consultant?

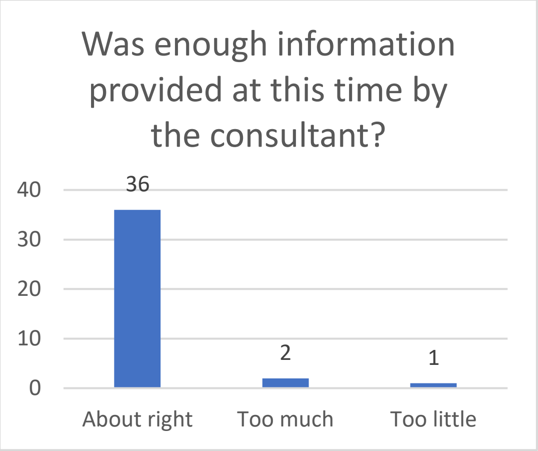

About right	36
Too much	2
Too little	1

Did you understand the consultant's information about Cystic Fibrosis at the time?

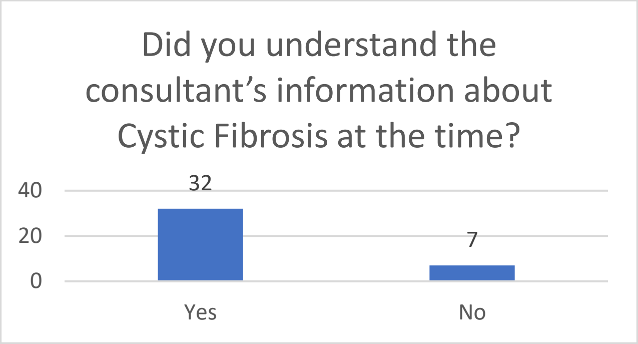

Yes	32
No	7

Did your child start any treatment the same day as the sweat test?

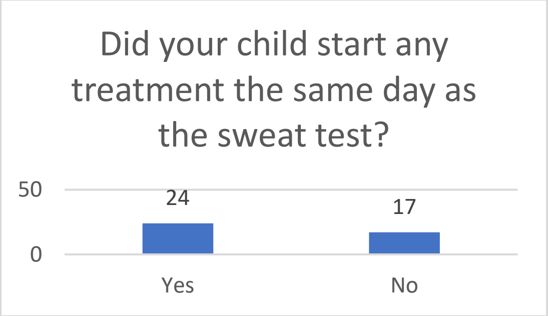

Yes	24
No	17

Creon?

Yes	21
No	1
Not answered	2

Antibiotics?

Yes	14
No	8
Not answered	2

If so did you feel you had enough training to feel comfortable in giving this treatment?

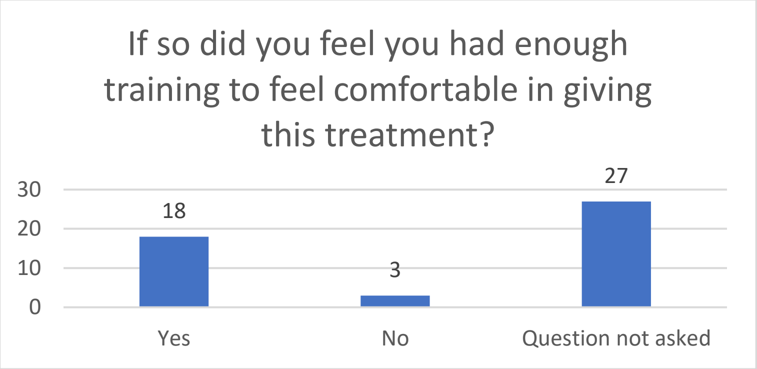

Yes	18
No	3
Question not asked	27

Can you think of any ways this part of the process could be improved?

A big part of coming to terms with the diagnosis was hope that one day there will be treatments & possibly a cure. Giving information about trials underway would I feel help with this
again, my only issue - and this will vary for everyone -but was having to go to London. It would have been better if the test could have possibly been held at a local hospital, but I understand that logistically this would be difficult.
Better facilities, more comfortable for a breastfeeding mum.
Consultant was excellent He spoke to us clearly about CF Spoke English rather than medical jargon
Everyone was amazing. We were looked after by *** who was very caring. *** explained everything very slowly and clearly. everyone was so helpful and gave us so much time.
I am not 100% sure when we met the consultant. It was packed, full on day, lots of information.
I came home with lots of bottles of medicine. I think I needed to give 8 syringes per day, including vitamins and having to draw these all up was overwhelming. .i think the home care nurse should visit the next day or two to see how things are going and give further advice.
I did but it all happened so quick we had to adapt very quickly. But for your child your prepared to do anything
I felt totally overwhelmed how to use syringes, how to make up a bottle of antibiotic, also i didn’t have a clue how to give it to him, and what times were the best especially as he was having such regular feeds and it’s hard to get time to give antibiotics on an empty stomach on a baby. I also had lots of questions and the doctor made me feel rushed and didnt answer many of them
I'm completely happy with how everything was dealt with
It was fine - I remember laughing at the size of the creon scoop. We felt everybody was trying to make the day as relaxed as possible and we came out of hospital feeling OK.
No areas for improvement - the team did a great job and were very patient. I found the information about CF (that *** was explaining) to be quite difficult to understand. But she was very patient and my partner grasped the information better and quicker (so my struggle was probably just because I was so tired and emotional). I think we started the

Creon on day 1, but I think the antibiotics and everything else started after the second day.
not really
Not really - our consultant (***) and the CNS (***) were both excellent Also - I can't remember when we met the consultant (sorry - but I can't untick the box above!)
Not really mine was a big blurred s my daughter got admitted straight away so
Possibly have a clinic set up for positive results so parents can have a starters course on how to manage initially caring for a baby with CF.
The consultant was brilliant. Very clear and excellent at answering questions.
The visit for the sweat test/result was very well organised. Consultant and specialist nurse took their time to explain what CF is, the test and the result
This day was really useful in terms of meeting the team and gathering more information prior to the education days
Too much info on day one especially about long term prospects. The picture painted was much darker than necessary and made a bad situation worse.
We waited a long time between *** having the sweat test and being told the results. It turned out that this was because ***'s results showed he was border line and they had to look back at the blood test results to get further information before coming back in to see us. It was better news than we had hoped but it was made clear that at such an early stage a lot could change over the years. Even though it was better than we had hoped for this was still tough to absorb as we had been preparing for something more serious and also more definite. Instead it felt a little unknown/unclear.
We were eager to begin treatment as *** had already been unwell, but he started on meds the day of the induction meeting which was just a day or two later.

Did you see the same consultant or nurse at the sweat test and at the education visit?

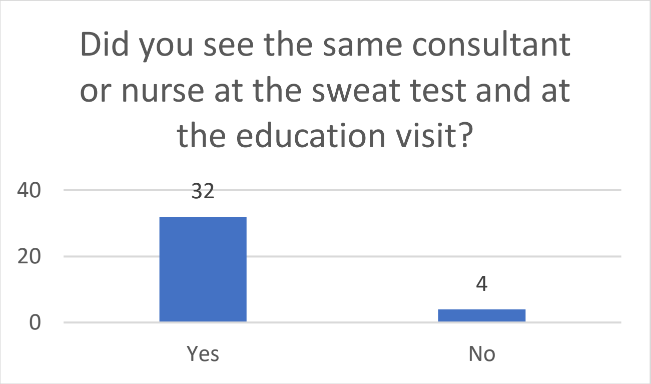

Yes	32
No	4

If no, would you have preferred to see the same professional? Please comment why

Not applicable to us. We did not require educational visit.
We saw *** at both visits which was really helpful.
We saw the same nurse, but a different consultant.

We were not invited to an education visit. Was this because *** wasn't officially diagnosed with CF at this stage, but instead he was put into the paed respiratory clinic? In hindsight, missing this education visit was a shame and has hindered our understanding of many things and consequently our overall experience. Looking back the education visit would've provided some clarification and crucial support that I feel we lacked in the first few years. Even though we regularly came to clinic, we saw different people each time. (Apart from *** who we've seen regularly, and *** at the beginning in each resp clinic but not since). The processes, our responsibilities, who to contact, dos and dont's etc have at times been identified by accident or even a little bit too late sometimes.
yes. I remember *** being present the whole way through, but I think the consultant / professor was different
Can't remember
Cant remember.
didn't matter
didn't really matter
It didn't matter that we saw different consultants.
It was nice to meet other members of staff.
It would have helped to build the relationship with the hospital. It was excellent that *** was consistently present at the hospital visits and that *** regularly visited and called me - this really helped to build the relationships, and it gave me a lot of reassurance about the quality of support that we would get from the team.
No. It was good to meet different members of the team
Not applicable to us. We did not require an educational visit.
The Consultant we saw for ***'s education is still her consultant now. We are very happy with him so glad it was a different doctor. The initial consultant was also very good and approachable, we had no issue with either consultant.
Yes. The consultant at the sweat test was much clearer and more helpful. Continuity here would have been reassuring

Was the level and (amount of detail) acceptable?

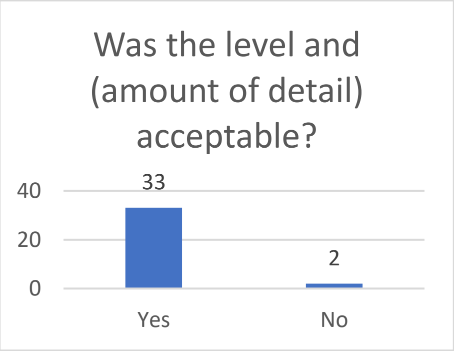

Yes	33
No	2

Comments received:

Can't remember

For that time there was more than enough detail. It was nice to meet all the professionals from each department separately and learn about the different aspects of cf and how we would manage each need as a family.
It was the correct level of information for the stage we were at - we were told the facts without being over loaded or made to worry about things that may not ever happen on the future
It's important to understand what CF is and what you have to do to keep your child safe. Important to meet all the person who would be involved in our child's care
Lots to take in but it was excellently done
Not applicable to us. We did not require educational visit.
The staff through the visit was very welcoming and helpfully through a very stressful upsetting time
There were no leaflets about things to avoid and these guidelines have only just been produced nearly 5 years later.
They were direct but compassionate about delivering the tough news. They did not try to sugar coat anything, yet they were compassionate - this is not an easy task and the whole team did really well.
We had a really long list of questions which they answered fully

Was there anything you would have wanted discussed that was not included?

How to administer oral syringes. We made a lot of mess initially!
No
No - not that I can remember
no but you don't know what questions to ask
no but you still are unaware of what questions to ask
No, everything was covered.
Not that I can remember.
See previous comment about trials/research
What to expect over the years How to practically fit in physio and antibiotics into a baby's day Cross infection and how to talk to the community How to take antibiotics and creon out and about for day trips etc What feelings to expect ie taking a baby to classes but having to clean the toys and people asking you why

Comments:

Also helped to have grandparents (we are lucky and have very supportive parents who help us lots!) invited
We spent a lot time talking about CF, what it is, how child future. Our child has a rare mutation of CF so we talked in length about what it meant for our child

Was a two-day visit?

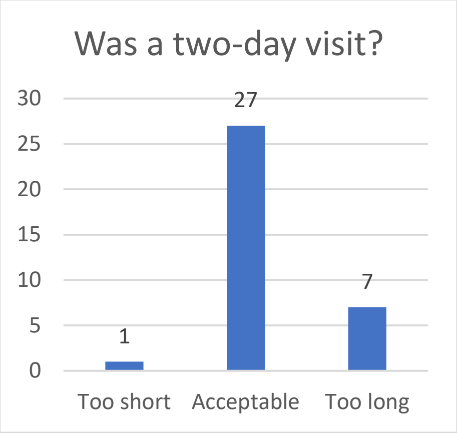

Too short	1
Acceptable	27
Too long	7

In your opinion, could you have had the same level of information given at a clinic visit or a one-day visit?

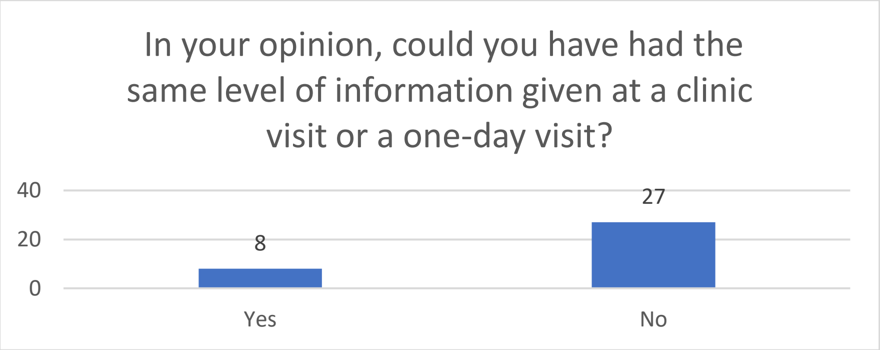

Yes	8
No	27

Comments received:

a one day would be much more intensive but cuts down on traveling into London and also the downtime at the Brompton between each chunk of the education visit
Again, due to the additional stress of travel, I would have much rather have completed this all in one day. I think we felt the second day (which I can't even remember!) really was necessary.
As parents (and grandparents) we needed that time to process all the information given. As the course is for 2 days this meant that we could go away and then talk about what we had learnt that evening and come up with questions for the next day.
As we had a baby and a 2 year old with us, 2 days felt too long (especially with very little sleep). You can't take in all of the information at first so I feel one day and then keep adding extra information at clinic visits would have been better.
Because we thought of more questions overnight. The timetable was v helpful.

I don't remember having a two day education visit. I think we had a one day visit but maybe I've misremembered.
I don't think we could have waited until next clinic appointment, I needed information as soon as possible
I remember there was a lot to take in & over 2 days helped to recheck on bit that we were not 100% sure on.
if a one day visit it would be more intensive but two days does involve a lot of traveling and some down time waiting at the Brompton
It felt far too long and the most useful was talking to the dietician and the psychologist.
It might have been possible to do it in less time, but we were really grateful to have a bit longer so that we could think about things, come up with questions etc. We lived very near at the time as well, so visiting was no problem.
It was very good to reflect on the information, read the CF Trust information and go back with clarifying questions the following day (as I certainly hadn't fully understood the information on the first day)
It was very useful to have day 1. digest and then attend again the next day. In terms on content it could have been covered in one day but we welcomed the two day support while digesting the diagnosis.
It would of been too overlaid with information
Not applicable to us. We did not require an educational visit.
Possibly - it would be a long day though (but would have been doable)
The days were long with a newborn and just too much information in one go.
There is too much information to take on board in one day and the 2nd day allows you to go away, digest the information and come back with any questions you may have
We had a crash course in 1 day as it was just before Christmas. A bit short as we got a lot of information quickly Hard to process everything
We had to digest the given information.
We spent most of the two days waiting for staff who showed up late or not at all. It was a terrible waste of time at a moment where we were very fragile

Would you have preferred the two-day education visit to be spread over a week? e.g. Tuesday one week and the following Monday the next

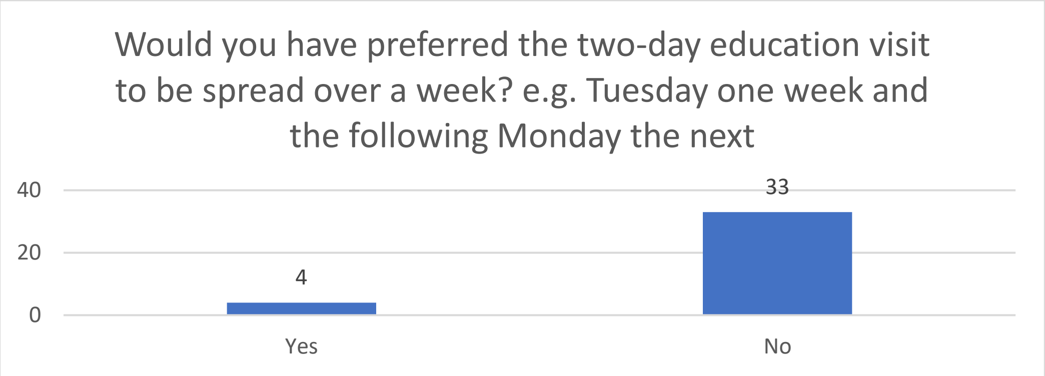

Yes	4
No	33

Are there any other improvements you think we could make to this part of the process?

3 years on I wish I could go back and tell myself that it's not the end of the world & to enjoy our beautiful baby girl. Being offered access to parents who have been through this & can tell you you will find a way through might have been useful
A one day visit followed by CF specialist nurse follow up at home.
Less waiting in between appointments!
Make it the next few days after diagnosis and be quicker, its hard to bring a baby into hospital all day
No
No this was very good, the timing was right and the information appropriate.
No, we found the education days good.
not really
Spreading the days so parents have time to think about what they learnt

Were you seen at home the following week by the Nurse Specialist?

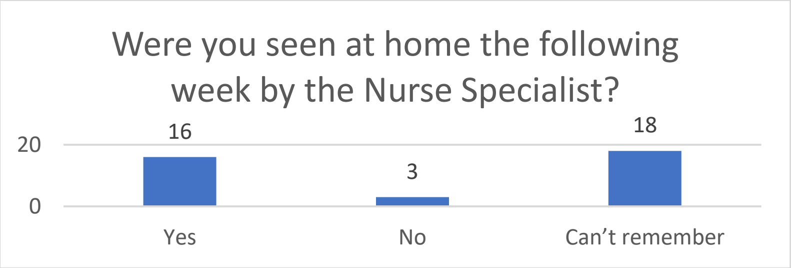

Yes	16
No	3
Can’t remember	18

If yes, was this useful?

Yes	15
No	1

Comments received:

It is great to have a follow up so you can ask the questions you didn't of asking before
It was a little over a week I think but this was not a problem at all
It was very useful to have a contact who I could call or text to ask anything.
*** has been incredible since day 1
My daughter was admitted so saw the nurses and consultants in hospital
The level of home support was excellent (far exceeded expectations). As a first time mum, I probably relied on ***’s support and her home visits more than I should have.
The week after receiving a home visit was very useful as it helped answer any questions we had.

Do you have any suggestions for improvements that could be made to this part of the process?

I think it would be helpful to look at the group of parents/children who transfer to a CF clinic later on and have therefore missed the standard introduction. Feel like we missed out on some really valid and useful information.
I think it would help if new parents were given written information about what clinic entails. I found often the people referred to clinic a lot, but at the time I didn't know what clinic involved. I also came to the first clinic really early and to the wrong entrance, so it would be helpful to have a written reminder when you're trying to take so much in. I was also a little confused about what to do if my son was ill... call the gp? go to the gp? call the hospital? call my nurse? was that only if he had a cold? what do I do if he has an upset tummy - is that cf related or a regular every day problem? I also didn't know how repeat prescriptions worked. It would help if parents understood the process of getting the letter to their gp. Their gp writing the prescription and getting the prescription to the pharmacy. It sounds very basic, but at the time I was in a haze and found it quite confusing.
It has improved since then, the appts now seem to run more smoothly than before.
Just a really long time to wait between seeing specialists. Do the cough swab first. alot of information was repeated and we didnt need to keep being told unless we ask
The only annoying bit of any clinic visit was waiting over an hour or two to collect prescriptions after seeing the consultant. By that point you just want to get home with your baby! If you saw the consultant first and got any prescription- it could be prepared for collection while you are seeing the other specialists on the team.

Do you know your child's CF genotype?

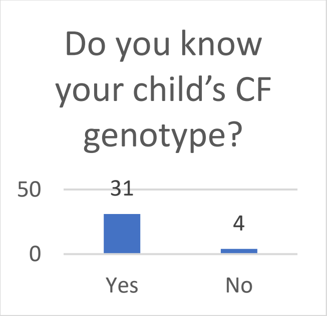

Yes	31
No	4

Can you remember when you were first told?

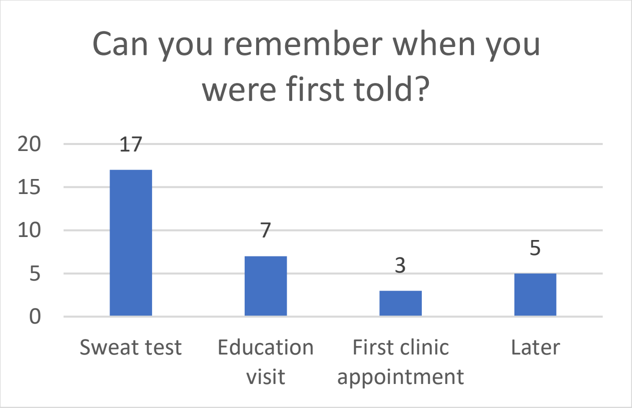

Sweat test	17
Education visit	7
First clinic appointment	3
Later	5

If we knew the genetic result (genotype) from the heel prick test indicated that your child had CF, should we have told you this at the home visit; or waited until proper confirmation of the diagnosis from the sweat test result?

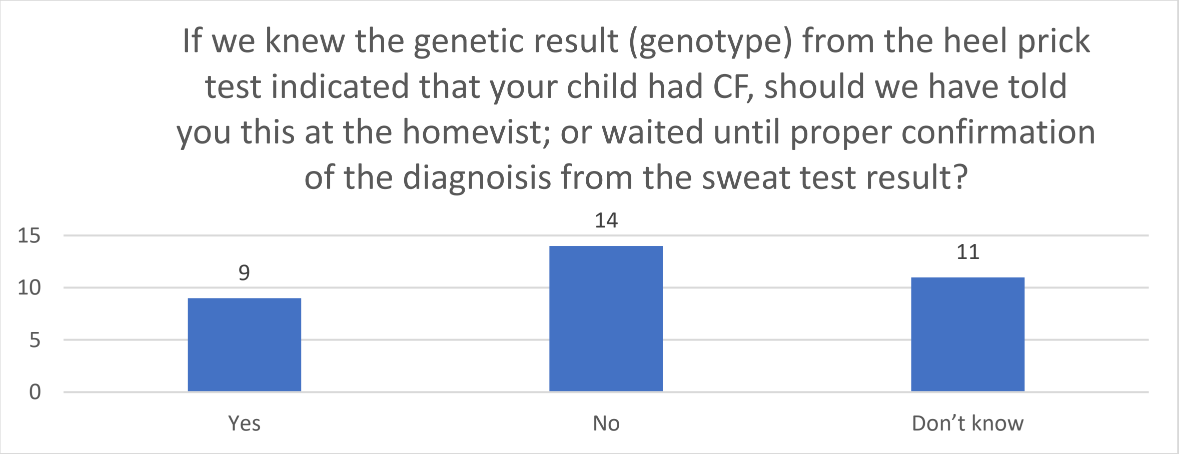

Yes	9
No	14
Don't know	11

Comments received:

Don't need that information until later really. It was fine finding out when we did.
I don't remember discussing the specific genotype so early. I think the information shared at the home visit could possibly be more "definite" (as per above, I was still hopeful that the heal prick test result was wrong and that there was a chance that the sweat test was the real test, and that there was a chance the result could come back negative).
I don't think this information would have been helpful for me. I'm sure I wouldn't have remembered it if I had been told.

I found it very frustrating that this information was withheld.
I think it was right to wait as we had a lot of information to get through at first and waiting gave us more knowledge to understand her genotypes
I think there is a lot to take in and waiting for the sweat test gives you some more time to process the situation and get your head round the possible diagnosis.
I would have liked to have known straight away.
if you definitely knew, we should be told immediately
It may have benefited us as our son has not (yet) developed CF but I don’t know if it would have benefitted others.
It was better to be told when it is confirmed
It's a lot of info to take on board at the home visit. Giving genetic info would've been lost on me.
No I think how they did it was perfect under the circumstance
Preferred to wait for the sweat test to know for sure
Probably unnecessary detail at this stage
Should have waited until sweat test result
The consultant is the right person to give this information.
The genotype would mean nothing to us at the home visit
Think you should wait
Waited for confirmation.
Waiting until the sweat test gives you hope that it might be wrong, if you already know from the genotype then I'd rather have been told at the home visit

Are there any other comments about the NBS service provided at the Royal Brompton Hospital that you would like to make?

Excellent
Great service and we are lucky to be under your care
I think the nurse who will be your homecare nurse should visit at the very start if that is at all possible.
I think they were brilliant & it was handled really well, it's a hard time & we felt supported
If NBS means processing the test results, it was v swift.
Nurse *** was amazing to us, very kind and sympathetic and helpful. Also *** and *** were very proactive, chatty and helpful to us. I wish that the clinics were in the mornings as by the evening the baby is more tired and irritable and clinics last too late in the day when it should be a baby's bedtime. We could tell that the time is stretched for every nurse/doctor/dietician etc and it felt rushed if we did need time to talk about things.
Overall a positive experience in difficult circumstances
The care shown to us when we first came up for the sweat test was second to none. Such a sensitive and daunting time for new parents and everyone we saw was so kind.
The process after the nurse visit was streamlined and I can't remember there being any problems. I feel the earlier process before we were given the first news needed some work. This is very serious and parents waiting for results is difficult. I would suggest better communication between the hospital doing the test and the health visitor could reduce the trauma of waiting. I also believe the person re-testing the blood should be more equipped to answer questions and not give vague answers. I actually thought the result must have been negative as it was taking so long for it to come through.

The service was amazing. This diagnosis was a shock, life changing and as a first time parent terrifying. Everything was organised for us and we had nothing to think about in this difficult time. The team is super professional and always took their time to explain .
The support provided by the team was excellent (and continues to be excellent), especially the doctors/professors, and *** and *** (our main CF nurses). I personally don't see the value in including the health visitor, as I never built up a relationship with mine (and I was often sent different HVs). Perhaps you could help to bring the doctors surgery into the loop a bit earlier, to provide support? I think the dieticians did a great job, especially in the early weeks/months - *** and *** were very proactive in their support (and very reactive, too) and were very encouraging. However the dieticians as a team can probably further improve their education around how to use creon, as the babies start the weening process.
the whole team have been fantastic and totally supportive over ***'s treatment. they have consistently answered questions, responded to calls and advised about meds and treatment always in a prompt and professional manor.
very friendly and professional - always available and respond to calls promptly allowing ***’s treatment to be adjusted accordingly
We couldn't wish for a better team of people at all
